# Hæmaturia in Tumours of the Bladder

**Published:** 1916-06-10

**Authors:** 


### Hæmaturia in Tumours of the Bladder.

Hæmaturia is the cardinal symptom, and the one which usually first attracts attention. This is true in 90 per cent. of the cases of benign papilloma and in 70 per cent. of malignant growths. As the bleeding is rarely accompanied by pain, its importance is apt to be overlooked by the patient, and thus assistance is often postponed to a relatively advanced stage. In respect to the absence of pain, bleeding from tumour differs from that caused by stone, and a similar contrast exists in regard to the effect of movement or exercise. The hæmaturia of tumour has no obvious relation to rest on the one hand or to activity on the other; it happens, as it were, without apparent cause. The quantity is often sufficient to give to the urine a bright red colour; it is usually specially abundant towards the end of micturition; and some of the blood may appear in the form of clots. In simple papilloma the bleeding is often more profuse than in carcinoma, and it appears at a much earlier stage of the disease; indeed, simple papilloma may give rise to even fatal hemorrhage. Multiple tumours in cystoscopic examination demand a serious interpretation, even though they exist merely as small warty growths. Similarly, pain in the bladder and radiating along the sciatic and crural nerves—the absence of cystitis being understood—carries a suggestion of malignancy. The difficulty of cystoscopic examination, due to mixture of blood with the introduced fluid, can be largely overcome if, instead of allowing the bladder to empty itself on irrigation, only half the fluid is permitted to escape and then more is introduced. By repeating this manœuvre several times a clear medium can frequently be obtained.—*Dr. David Newman, in the "Glasg. Med. Journ."*

